# Falling charge in a gravitational field and radiation reaction

**DOI:** 10.1038/s41598-024-54731-4

**Published:** 2024-02-29

**Authors:** Paul Bracken

**Affiliations:** Department of Mathematics, University of Texas, Edinburg, TX 78540 USA

**Keywords:** Charge, Gravity, Relativity, Electrodynamics, Time, Tensor, Mathematics and computing, Physics

## Abstract

The Lorentz–Dirac equation is formulated and studied in flat Minkowski spacetime. A concise, novel derivation of the equation is presented. The problem is then enlarged to study radiation damping of an electron moving through a gravitational field. The equation of motion is obtained for this case as well. It is suggested the study of the problem might motivate experiments which could shed light on the recent work related to the emergence of space-time and its structure by means of quantum effects such as quantum entanglement.

## Introduction

It is well known that charges radiate when they undergo acceleration^1^. This fact was a major motivation for the development of quantum mechanics when the structure of atoms required electrons to circulate about a nucleus. In this picture electrons are restricted to orbitals around a nucleus. Since electrons in such orbitals can be thought to accelerate, they should radiate due to their confined localized motion. Classically then this means they accelerate and thus radiate energy. This does not take place, as atoms remain stable, and indicates that new physical laws, those of quantum mechanics, must be adopted to account for the structure of atoms. Accelerations are also produced as a result of the curvature of space-time. This curvature can result from a large concentration of mass such as that associated with a star. The curvature about this mass distribution is determined by Einstein’s equations. A mass distribution in some region of space-time is responsible for this curvature which creates the gravitational field. To study how electromagnetism and gravity are related, the problem of the falling charge treated classically in such a field may provide some information as to physics and gravity. This problem has been investigated classically before by DeWitt and Brehme as well as by Hobbs^2–5^. It would be worth returning to the idea in the hope of suggesting new experiments which could shed new light on current theories of spacetime. For example, the emergence of classically connected spacetimes has been proposed to be intimately related to the quantum entanglement of quantum particles. This might be the case since the radiation which is produced has a number of distinguishing characteristics.

The principle of equivalence which has been verified for neutral matter and the principle of covariance of physical laws constitute the basis for the general theory of relativity. Whether a falling charge radiates is like asking does the equivalence principle apply to charged matter. Originally the principle was never meant to apply other than in a local way to physical objects undergoing acceleration. A classical charge however is not strictly a local object when the Coulomb field is taken into account. The principle of equivalence is a local relationship which states that a gravitational force cannot be distinguished from a force that is inertial by means of an experiment carried out locally.

The solution and study of the covariant scalar and vector wave equations

1 $$\begin{aligned} g^{\mu \nu } \, \phi _{; \mu \nu } =0, \qquad g^{\nu \sigma } A_{\mu ; \nu \sigma } + R_{\mu }^{\nu } A_{\nu } =0, \end{aligned}$$

was initiated by Hadamard^6^. He tried to find a so-called elementary solution which, in the case of a 4-dimensional space-time is a bi-scalar of the form

2 $$\begin{aligned} G^{(1)} = \frac{1}{(2 \pi )^2} \, \left( \frac{u}{\sigma } + v \, \ln |\sigma | + w \right) , \end{aligned}$$

where *u*, *v* and *w* are bi-scalars which are free of singularities and satisfy the normalization condition $\lim _{x \rightarrow z} \, u =1$.

Here the objective is to start off by reviewing Dirac’s 1938 paper on the classical radiating electron^7^ in which proceeds in a Lorentz covariant way. Next a development of the radiating falling charge problem is presented^8^ which is hopefully concise and more comprehensible. Consider a charge in free space in a region with nonzero curvature and not near the masses generating it. When subjected to a force it accelerates and radiates a reactive damping force in addition to its mechanical inertial force. It might be thought a charge placed in a static gravitational field does not radiate under these conditions. However, an accelerated charge does not suffer a reactive damping force if its absolute acceleration is uniform. If a particle is far from a gravitating object it may be thought to be in a state of uniform motion and does not radiate. If it moves into a region with a strong gravitional field the motion of the unaccelerated particle changes to a state of free fall, and it seems reasonable to say that a charged particle radiates as it moves through a region with a gravitational field.

The potential is the spacetime metric in general relativity and second derivatives of the metric tensor are involved in the Riemann tensor which describes the intrinsic space-time curvature or gravitational field. It can be said the charged particle attempts to satisfy the equivalence principle and it manages to do so locally. The particle however deviates from geodetic motion due to the Coulombic tail in the propagator function for the electromagnetic field. This enters nonlocally by appearing as an integral term over the past history of the particle. The Lorentz–Dirac equation in Minkowski spacetime is introduced here for a relativistic charge. This equation can be generalized without using a special coordinate system at any stage to the case in which the background spacetime is curved. In so doing, it is found that a tail function in the presence of nonzero curvature appears in the result^7–10^. The model is purely classical. However, the physical impact of the quantum vacuum is briefly mentioned at the end^11–14^. Different types of vacuum can exist. Unruh has shown that a uniformly accelerated observer whose acceleration is $\alpha$, the Minkowski vacuum takes the appearance of a thermal state at a temperature $\textbf{a}/ 2 \pi$. The charge sees the vacuum fluctuations as comoving and comprise a thermal bath. For a charge constrained to move with constant acceleration, there can be no net transfer of energy momentum between charge and vacuum as observed from the accelerated frame.

## Electrodynamics in Minkowski space

An electromagnetic field $F_{\alpha \beta }$ is generated by a charge *e* moving through a flat space-time on a world line $z^{\alpha } (\tau )$, where $\tau$ is the proper time associated with the particle. In what follows, the four-velocity $u^{\alpha } (\tau )$ and acceleration $a^{\alpha } (\tau )$ of the charge are defined as

$$\begin{aligned} u^{\alpha } (\tau ) = \frac{d z^{\alpha }}{d \tau }, \qquad a^{\alpha } ( \tau ) = \frac{d u^{\alpha }}{d \tau }. \end{aligned}$$

The current $j^{\mu } (x)$ associated with the charge is given by

3 $$\begin{aligned} j^{\mu } (z) = e \, \int \, dz \, u^{\alpha } \delta ( x - z). \end{aligned}$$

The current $j^{\mu }$ appears in the field equations for the particle

4 $$\begin{aligned} \Box \, A^{\alpha } = - 4 \pi j^{\alpha }, \qquad \Box = \eta ^{\alpha \beta } \, \partial _{\alpha } \partial _{\beta }. \end{aligned}$$

A general solution of this equation is can be given by using the Green function $G (x, x')$ for the operator $\Box$ in (4),

5 $$\begin{aligned} \Box \, G (x, x') = - 4 \pi \delta (x- x'). \end{aligned}$$

If $A^{\alpha }_{hom}$ is any solution to the homogeneous equation then

6 $$\begin{aligned} A^{\alpha } (x) = \int \, G (x,x') \, j^{\alpha } (x') \, d^4 x' + A^{\alpha }_{hom} (x). \end{aligned}$$

In (6) *G* is the retarded Green’s function. The retarded and advanced Green’s functions are defined to be

7 $$\begin{aligned} \begin{array}{c} G_{ret} (x,x') = \displaystyle \frac{\delta (t-t'- | \textbf{x} - \textbf{x}'|)}{|\textbf{x} - \textbf{x}'|}. \\ \\ G_{adv} ( x, x') = \displaystyle \frac{\delta ( t- t' + |\textbf{x} - \textbf{x}'|)}{| \textbf{x} - \textbf{x}'|}. \\ \end{array} \end{aligned}$$

To calculate the potential and field fully relativistically for a charge, the causal structure of the retarded Green’s function $G_{ret} ( x,x')$ supported on the past light cone of the field point *x* is needed. Of course the field at *x* depends on the state of motion of the charge exactly where the world line intersects the past light cone. The past light cone can be used as a natural mapping between the field point *x* and a specific point *z* on the world line. Thus let *x* be the field point, *z*(*u*) the point where the world line intersects the past light cone of *x*. Thus *u*(*x*) denotes the specific value of the particle’s proper time $\tau$ corresponding to the intersection point. Thus *u* may be called the retarded time determined by *x*.

8 $$\begin{aligned} \frac{1}{2} \eta _{\alpha \beta } |x^{\alpha } - z^{\alpha } (u) | \cdot | x^{\beta } - z^{\beta } (u) | =0. \end{aligned}$$

This implies *x* and *z* (*u*) are joined by a null geodesic. An invariant measure of the distance between *x* and *z* suggests that we consider the scalar quantity *r*(*x*) defined as

9 $$\begin{aligned} r (\textbf{x}) =- \eta _{\alpha \beta } [ x^{\alpha } - z^{\alpha } (u) ] \, u^{\beta } (u). \end{aligned}$$

Since *c* has been set to one, *r* in (9) is the spatial distance between these two points. It is referred to as the retarded distance between the field point and the particle. As *u* is determined by *x* in (9), there is no need to make the dependence on *u* explicit so we write $r ({x}) = r ({x}, u)$. The vector ${x} - {z} (u)$ is a null vector pointing from *z* (*u*) to *x*, and may be rescaled by the factor 1/*r* as,

10 $$\begin{aligned} k^{\alpha } ({x}) = \frac{1}{r} \, [ x^{\alpha } - z^{\alpha } ( \eta ) ]. \end{aligned}$$

Moreover $k^{\alpha }$ in (10) satisfies

11 $$\begin{aligned} k_{\alpha } ({x}) k^{\alpha } ({ x})=0, \qquad k_{\alpha } ({x}) u^{\alpha } ({x}) =-1. \end{aligned}$$

The retarded solution to the field equation (4) with current density (3) is,

12 $$\begin{aligned} A^{\alpha } (\textbf{x}) = e \, \int d \tau \, u^{\alpha } G_{ret} ({ x}, {z}). \end{aligned}$$

To evaluate the integral in (12), a change of variable is made. We introduce $d \tau = d \sigma / \dot{\sigma }$ and since $\sigma$ runs over negative to positive values as $\tau$ goes through the retarded time *u*, $\dot{\sigma }$ is positive when $\tau =u$. In fact $\dot{\sigma } (\tau = u)= r ({x})$. The vector potential in (11) is given by

13 $$\begin{aligned} A^{\alpha } ({x}) = e \frac{u^{\alpha } (u)}{r ({x})}. \end{aligned}$$

This is usually called the Liénard–Weichert potential.

The electromagnetic field $F_{\alpha \beta }$ is obtained from the scalar potential by regarding $\textbf{z}$ and $\eta$ to be independent variables and setting $f (\textbf{x})= F (\textbf{x}, {u})$ under the light cone mapping. Suppose $\textbf{x}$ is displaced to $\textbf{x} + \delta \textbf{x}$ so the new intersection point is $\textbf{z} ( {u} + \delta {u})$. These points are related by the condition $\sigma ( \textbf{x} + \delta \textbf{x}, {u}+ \delta {u})=0$. Expanding this to first order in $\delta x, \delta u$ and using (11) gives the result $k_{\alpha } \, \delta x^{\alpha } + \delta {u}=0$ or

14 $$\begin{aligned} \frac{\partial u}{\partial x^{\alpha }} =- k_{\alpha }. \end{aligned}$$

By means of (14),

15 $$\begin{aligned} \frac{\partial f}{\partial x^{\alpha }} = \left( \frac{\partial F}{\partial x^{\alpha }} \right) _u - k_{\alpha } \left( \frac{\partial F}{\partial \eta } \right) _x. \end{aligned}$$

This is the differentiation rule under the light cone mapping. An application of (15) is to get the derivative of the retarded distance which we simply call $r_{\alpha }$,

16 $$\begin{aligned} r_{\alpha } = r_{, \alpha }= - u_{\alpha } + ( 1 + r a_k ) \, k_{\alpha }. \end{aligned}$$

where $a_k = a_{\alpha } k^{\alpha }$ is the component of the acceleration $a^{\alpha }$ in the direction of $k^{\alpha }$. We also have $k_{\alpha } (x) r^{\alpha } (x) =-1$. Using $u_{\alpha , \beta } =- a_{\alpha } k_{\beta }$ and (16), we obtain using $a_k = a_{\alpha } k^{\alpha }$ that

17 $$\begin{aligned}{} & {} F_{\alpha \beta } = A_{\beta , \alpha } - A_{\alpha , \beta } = e \left( \frac{u_{\beta , \alpha } (x)}{r(x)} - \frac{u_{\beta } (x)}{r (x)^2} r_{, \alpha } - \frac{u_{\alpha , \beta } (x)}{r (x)} + \frac{u_{\alpha } (x)}{r(x)^2} r_{, \beta } \right) \nonumber \\{} & {} \quad = e \left( - \frac{a_{\beta } k_{\alpha }}{r(x)} - \frac{u_{\beta } (x)}{r(x)^2} ( - u_{\alpha } + ( 1 + r a_k) k_{\alpha }) + \frac{a_{\alpha } k_{\beta }}{r(x)} + \frac{u_{\alpha } (x)}{r(x)^2} (- u_{\beta } + ( 1 + r a_k) k_{\beta } \right) \nonumber \\{} & {} \quad = \frac{2 e}{r} ( a_{[ \alpha } k_{\beta ]} + a_k \, u_{[\alpha } k_{\beta ]} ) + \frac{2 e}{r^2} \, u_{[\alpha } k_{\beta ]}. \end{aligned}$$

The dependence of the world line quantities on the retarded time *u* has been suppressed. Square brackets in (17) denote antisymmetrization,

18 $$\begin{aligned} a_{[ \alpha } \, k_{\beta ]} = \frac{1}{2} ( a_{\alpha } k_{\beta } - a_{\beta } k_{\alpha } ). \end{aligned}$$

The energy-momentum tensor for the electromagnetic field is obtained by substituting $F_{\alpha \beta }$ into it

19 $$\begin{aligned} T^{\alpha \beta }_{em} = T^{\alpha \beta }_{rad} + T^{\alpha \beta }_{bnd}. \end{aligned}$$

In (19), the first term is the radiation component

20 $$\begin{aligned} T^{\alpha \beta }_{rad} = \frac{e^2}{4 \pi r^2} \big ( a^2 - (a^{\alpha } k_{\alpha })^2 \big ) k^{\alpha }, k^{\beta }, \end{aligned}$$

where $a^2 = a_{\alpha } a^{\alpha }$ and the Coulomb or bound component $T_{bnd}$ is given by

21 $$\begin{aligned} T^{\alpha \beta }_{rad} = \frac{e^2}{2 \pi r^3} [ k^{(\alpha } a^{\beta )} + a_k ( k^{(\alpha } u^{\beta )} - k^{\alpha } k^{\beta })] + \frac{e^2}{2 \pi r^4} \left[ 2 k^{(\alpha } u^{\beta )} - k^{\alpha } k^{\beta } + \frac{1}{2} \eta ^{\alpha \beta }\right] . \end{aligned}$$

The decomposition (19) has the following properties for $T^{\alpha \beta }_{rad}$ and $T^{\alpha \beta }_{bnd}$ when $r \ne 0$,

22 $$\begin{aligned} \partial _{\beta } \, T^{\alpha \beta }_{rad}=0, \qquad \partial _{\beta } \, T^{\alpha \beta }_{bnd} =0. \end{aligned}$$

The interpretation of $T^{\alpha \beta }_{rad}$ as the radiation part of the stress-energy tensor is motivated by the fact that it scales as $r^{-2}$ and it is proportional to $k^{\alpha } k^{\beta }$.

## The Lorentz–Dirac equation

The Lorentz–Dirac equation of motion for a charged particle under the influence of an external force as well as its own electromagnetic field is developed in flat space first. The world line of the particle is given by $z^{\alpha } ( \tau )$. This gives the particle’s world line coordinates as a function of proper time.

Dirac’s derivation is based on consideration of energy-momentum conservation. The world-line of the charge can be placed within the world tube $\Sigma$. This can for example be a three cylinder in three space projected through time. It is desired to calculate how much electromagnetic field momentum flows across the surface per unit time. A change in the momentum flow must be balanced by a corresponding change in the particle momentum, so the total momentum is conserved.

The flux of the four-momentum across $\Sigma$ is given by

23 $$\begin{aligned} \Delta p^{\alpha } = \int _{\Sigma } \, T^{\alpha \beta } \, d \Sigma _{\beta }. \end{aligned}$$

In (23), $T^{\alpha \beta }$ is any conserved stress-energy tensor, $d \Sigma _{\beta }$ the outward directed element on $\Sigma$. If $\Sigma '$ is a deformation of $\Sigma$, the momentum flow through the deformed tube $\Sigma '$ is the same as through $\Sigma$ provided the two tubes begin and end on the same two-surface. To see this, let $\Delta p^{\alpha '}$ be the momentum flow across $\Sigma '$. Let ${{\mathcal {V}}}$ be the four-dimensional region between $\Sigma$ and $\Sigma '$ with boundary $\partial {{\mathcal {V}}}$ which consists of the union $\Sigma \cup \Sigma '$. By the theorem of Gauss

24 $$\begin{aligned} \Delta \, p^{\alpha '} - \Delta \, p^{\alpha } = \int _{\partial {\mathcal {V}}} \, T^{\alpha \beta } \, d \Sigma _{\beta } = \int _{{\mathcal {V}}} \, T^{\alpha \beta }_{, \beta } \, d V. \end{aligned}$$

As the shape is irrelevant, let $\Sigma$ be a hypersurface of constant *r*. Suppose *r* is small enough so the region lies close to the world line of the particle and $d \Sigma _{\alpha } = r_{\alpha } r^2 du d \, \Omega$ is the outward directed surface element of a three-cylinder with *r* constant in Minkowski space. Also $r_{\alpha } = \partial r/ \partial x^{\alpha }$ is the gradient of *r* in any coordinate system $x^{\alpha }$.

From (20), the radial component of $T^{\alpha \beta }_{rad}$ is

$$\begin{aligned} T^{\alpha \beta }_{rad} r_{\beta } = \frac{q^2}{4 \pi r^2} ( a^2 - a_k^2 ) \, k^{\alpha }. \end{aligned}$$

Consequently, the flow of the radiative momentum is

25 $$\begin{aligned} \Delta P^{\alpha }_{rad} = \frac{q^2}{4 \pi } \int \, ( a^2 - a_k^2) k^{\alpha } \, du d \Omega . \end{aligned}$$

Note that $r^2$ has cancelled out and (25) yields the rate of momentum change

26 $$\begin{aligned} \frac{d P^{\alpha }_{rad}}{d u} = \frac{q^2}{4 \pi } \int \, ( a^2 - a_k^2) \, k^{\alpha } \, d \Omega = \frac{2}{3} q^2 a^2 \, u^{\alpha }, \end{aligned}$$

since

27 $$\begin{aligned} \int \, k^{\alpha } \, d \Omega = 4 \pi u^{\alpha }, \qquad \int \, k^{\alpha } k^{\beta } \, d \Omega = 4 \pi \big ( \frac{1}{3} g^{\alpha \beta } + \frac{4}{3} u^{\alpha } u^{\beta } \big ). \end{aligned}$$

Using these integrals and $(u \cdot a)=0$ we find that

$$\begin{aligned} \frac{d P_{rad}^{\alpha }}{d u} = e^2 \left( a^2 u^{\alpha } - \frac{1}{3} (( a \cdot u) a^{\alpha } + a^2 u^{\alpha } ) + 2 (u \cdot a)^2 u^{\alpha } \right) = \frac{2}{3} e^2 a^2 \, u^{\alpha }. \end{aligned}$$

This is the amount of radiative momentum crossing a surface *r* equal to a constant per unit proper time.

The radial component of the bound stress-energy tensor is given by

28 $$\begin{aligned} T^{\alpha \beta }_{bnd} \, r_{\beta } = \frac{e^2}{4 \pi r^2} \, \left[ a^{\alpha } + a_k ( u^{\alpha } - \frac{3}{2} k^{\alpha }) \right] + \frac{e^2}{4 \pi r^4} ( u^{\alpha } - k^{\alpha } ) \end{aligned}$$

Using (27), the most singular terms at the end disappear upon integration, but the first term yields a non-zero result,

29 $$\begin{aligned} \frac{d P^{\alpha }_{rad}}{d u} = \frac{e^2}{2 r} \, a^{\alpha }. \end{aligned}$$

This is the rate of change of the bound momentum.

The rate of change of electromagnetic momentum is obtained by combining these two derivatives setting $e^2/ 2r = m_{em}$

30 $$\begin{aligned} \frac{d p_{em}^{\alpha }}{d u} = \frac{e^2}{2r} a^{\alpha } + \frac{2}{3} e^2 a^2 u^{\alpha } = m_{em} a^{\alpha } + \frac{2}{3} e^2 a^2 u^{\alpha }. \end{aligned}$$

It is now required that the total momentum $p_{em}^{\alpha } + p_{mech}^{\alpha }$ be conserved

31 $$\begin{aligned} \frac{d}{d u} ( p_{em}^{\alpha } + p_{mech}^{\alpha } ) =0. \end{aligned}$$

In (31), $p_{mech}^{\alpha }$ is the mechanical momentum of the particle itself. The idea is to identify the correct relation between $p_{mech}$ and the world-line quantities, so (31) becomes an equation of motion. To do this, another hypothesis is required. If one identifies $p_{mech}^{\alpha } = m_0 \, u^{\alpha }$, with $m_0$ the particle’s material mass, one obtains $a_{\alpha }$ proportional to the four-velocity. This is a nonsensical result because the acceleration cannot be proportional to the four-velocity. If *C* is a dimensionless constant, a reasonable choice is

32 $$\begin{aligned} p_{mech}^{\alpha } = m_0 \, u^{\alpha } + C e^2 a^{\alpha }. \end{aligned}$$

Combining (32) with (30) and (31), we obtain

$$\begin{aligned} m_{em} a^{\alpha } a^{\alpha } + \frac{2}{3} e^2 a^2 u^{\alpha } = \frac{ d p^{\alpha }_{em}}{d u} =- \frac{d p_{mech}^{\alpha }}{d u} = - m_0 a^{\alpha } - C e^2 \dot{a}^{\alpha }. \end{aligned}$$

Solving for $a^{\alpha }$ and setting $m=m_0+m_{em}$

33 $$\begin{aligned} m a^{\alpha } =- e^2 ( C \, \dot{a}^{\alpha } + \frac{2}{3} a^2 u^{\alpha } ). \end{aligned}$$

If the right-hand side of (33) is to be orthogonal to $u^{\alpha }$, then with the identity $a^2= - \dot{a}^{\alpha } \, u_{\alpha }$, it must be that $C = -2/3$. Combining these results yields the Lorentz–Dirac equation

34 $$\begin{aligned} m a^{\alpha } = \frac{2}{3} \, e^2 \left( \dot{a}^{\alpha } - a^2 \, u^{\alpha } \right) . \end{aligned}$$

Here *m* is the physical mass of the particle, the material contribution plus the electromagnetic contribution.

## Radiation damping of the electron in a gravitational fields

The objective is to derive the radiation damping term in the equation of motion in a curved space. The equation of motion of an electron in a curved space has been studied by DeWitt and Brehme^4^ as well as Hobbs^5^ by generalizing Dirac’s procedure. This is done by computing the flux of the energy-momentum tensor across a thin tube that encloses the particle’s world line. Starting from the equation of motion

35 $$\begin{aligned} m \ddot{z}_{\alpha } = e F^{in}_{\alpha \beta } \, \dot{z}^{\beta }, \end{aligned}$$

the idea is to apply a kind of analytic prolongation to (35) taking into account the retarded field. This is done by writing (35) as

36 $$\begin{aligned} m_0 \ddot{z}^{\mu } ( \tau + \tau _0) = e [ F^{in \, \mu }_{\nu } ( x= z (\tau + \tau _0)) + F^{\mu }_{\nu } (x= z ( \tau + \tau _0)), z ( \tau )] \dot{z}^{\nu } ( \tau + \tau _0) \end{aligned}$$

Then (36) is expanded in powers of the small parameter $\tau _0$. The divergent term that appears when $\tau _0 \rightarrow 0$ is absorbed by a renormalization procedure similar to that used to arrive at (34). The equation of motion follows by taking the limit $\tau _0 \rightarrow 0$. It is shown the equation of motion for the electron can be obtained in a Riemannian space this way. In a general space, covariance under arbitrary transformations of the coordinates must be maintained.

The characteristic function or world function $\sigma ( x,z)$ admits covariant expansions. The geodesic interval *s* gives the magnitude of the invariant distance between *x* and *z* as measured along the geodesic joining them. The world function is defined as

37 $$\begin{aligned} \sigma = \pm \frac{1}{2} \, s^2. \end{aligned}$$

It is positive for space-like intervals and negative for time-like intervals. Indices $\alpha$ to $\kappa$ are always associated to point *z*, and $\lambda$ to $\omega$ are associated with point *x*. The electron world line is given by a set of functions $z^{\alpha } ( \tau )$ where $\tau$ is the proper time. Dots over *z* denote absolute covariant differentiation with respect to $\tau$ in the following context,

38 $$\begin{aligned} \begin{array}{c} \dot{z}^{\alpha } = \displaystyle \frac{d z^{\alpha }}{d \tau }, \qquad \ddot{z}^{\alpha } = \displaystyle \frac{d \dot{z}^{\alpha }}{d \tau } + \Gamma ^{\alpha }_{\beta \gamma } \dot{z}^{\beta } \dot{z}^{\gamma } \\ \\ \dot{z}^{\alpha } \dot{z}_{\alpha } =-1, \qquad \dot{z}^{\alpha } \ddot{z}_{\alpha } =0. \\ \end{array} \end{aligned}$$

or semicolen otherwise. The field $F_{\mu \nu }$ is a bi-tensor that depends on the point *x* where the field is evaluated and on *z* which is the retarded point associated with *x* and is defined by

39 $$\begin{aligned} \sigma ( x, z ( \tau _{-})) =0. \end{aligned}$$

DeWitt^4^ also introduces a bi-scalar $\Delta$ deined $\Delta = {\bar{g}}^{-1} D$ where *D* and ${\bar{g}}$ are the following determinants

$$\begin{aligned} D=- | - \sigma _{\alpha \mu } |, \qquad {\bar{g}} = - | {\bar{g}}_{\alpha \mu } | \end{aligned}$$

The world function $\sigma ( z,x)$ associated to points *x*, *z* is the standard element on which expansions can be based. For a two tensor $T_{\alpha \beta }$ whose indices refer to the same point *z*, and admits the expansion in covariant form

40 $$\begin{aligned} T_{\alpha \beta } (x,z) = A_{\alpha \beta } + A_{\alpha \beta }^{\gamma } \sigma _{; \gamma } + \frac{1}{2} A_{\alpha \beta }^{\gamma \delta } \, \sigma _{; \gamma } \sigma _{; \delta } + O (s^3) \end{aligned}$$

where *s* is the expansion joining *x* and *z*. The coefficients in (40) are ordinary local tensors at *z*. They can be determined from the equations

41 $$\begin{aligned} \sigma _{; \mu } \sigma ^{; \mu } = \sigma _{; \alpha } \sigma ^{; \alpha } = 2 \, \sigma . \end{aligned}$$

If a bi-tensor exists whose indices do not all refer to the same point *z*, such as the field $F_{\mu \nu }$, define a new tensor all of whose indices do refer to point *z*, with the help of the bi-vector of geodesic parallel displacement ${\bar{g}}_{\mu \alpha }$ and expand the new tensor by means of (40).

As (36) indicates, the field $F_{\mu \nu }$ must be evaluated at $x= z ( \tau + \tau _0)$. The point $z (\tau )$ is not the retarded one associated with *x*. The world function that appears in the expansions is associated with the geodesic that joins the points $x = z (\tau + \tau _0)$ and $z ( \tau )$ with expansion parameter $\tau _0$. Due to this reason it is necessary to find the coefficients $A_{\alpha }, B_{\alpha }, \ldots$ which appear in the following expansion

42 $$\begin{aligned} \sigma _{; \alpha } ( x ( \tau + \tau _0), z ( \tau )) = A_{\alpha } \tau _0 + B_{\alpha } \tau _0^2 + C_{\alpha } \tau _0^3 + O ( \tau _0^4). \end{aligned}$$

The coefficients $A_{\alpha }, B_{\alpha }, \ldots$ are local vectors at $z ( \tau )$. To this end, consider the following quantity

43 $$\begin{aligned} \sigma _{; \alpha } ( x ( \tau + \tau _0), z ( \tau ^*)) = \sigma _{; \alpha } + \dot{\sigma }_{\alpha } \tau _0 + \frac{1}{2} \ddot{\sigma }_{; \alpha } \tau _0^2 + \frac{1}{6} \dddot{\sigma }_{; \alpha } \tau _0^3 + O ( \tau _0^4). \end{aligned}$$

The geodesic of the world function on the left-hand side is the one that joins $x ( \tau + \tau _0)$ and $z ( \tau ^*)$. The corresponding geodesic on the right the one joining $x (\tau )$ and $z (\tau ^*)$. As the derivatives $\dot{\sigma }_{\alpha }$, $\ddot{\sigma }_{\alpha }$ are evaluated at $x ( \tau )$ in covariant form, they are

44 $$\begin{aligned} \dot{\sigma }_{\alpha } = \sigma _{; \alpha } \dot{x}^{\mu }, \qquad \ddot{\sigma }_{; \alpha } = \sigma _{; \alpha \mu \nu } \dot{x}^{\mu } \dot{x}^{\nu } + \sigma _{; \alpha \mu } \ddot{x}^{\mu }, \qquad \dddot{\sigma }_{\alpha } = \sigma _{; \alpha \mu \nu \omega } \dot{x}^{\mu } \dot{x}^{\nu } \dot{x}^{\omega } + 3 \sigma _{; \alpha \mu \nu } \ddot{x}^{\mu } \dot{x}^{\nu } + \sigma _{; \alpha \mu } \dddot{x}^{\mu }. \end{aligned}$$

The coefficients of (39) can be found from the following results

$$\begin{aligned} \lim _{x \rightarrow z} \, \sigma _{; \alpha } =0, \qquad \lim _{x \rightarrow z} \, \sigma _{; \alpha \mu } = - g_{\mu \alpha }, \qquad \lim _{x \rightarrow z} \, \sigma _{; \alpha \mu \nu } =0, \end{aligned}$$

45 $$\begin{aligned} \lim _{x \rightarrow z} \, \sigma _{; \alpha \mu \nu \omega } = \lim _{x \rightarrow z} \, {\bar{g}}^{\tau }_{\alpha } \, \left( \frac{2}{3} R_{\tau \nu \mu \omega } - \frac{1}{3} R_{\tau \omega \mu \nu } \right) \end{aligned}$$

Letting *x* approach *z* in (43) and using (44) and (45) as well as symmetries of the Riemann tensor,

46 $$\begin{aligned} \sigma _{\mu \alpha } ( x ( \tau + \tau _0), z ( \tau )) = - \dot{z}_{\alpha } \tau _0 - \frac{1}{2} \ddot{z}_{\alpha } \tau _0^2 - \frac{1}{6} \dddot{z}_{\alpha } \, \tau _0^3 +O( \tau _0^4). \end{aligned}$$

From this equation and (38), it follows that

47 $$\begin{aligned} \kappa = \dot{\sigma }_{; \alpha } ( x ( \tau + \tau _0), z (\tau )) \, \dot{z}^{\alpha } (\tau ) = \left( 1 + \frac{1}{6} \ddot{z}^2 \tau _0^2 + O ( \tau _0^3)\right) \tau _0. \end{aligned}$$

Following a similar procedure, we can also define

48 $$\begin{aligned} \tilde{\kappa } = \sigma _{; \alpha } \ddot{z}^{\alpha } =- \frac{1}{2} \ddot{z}^2 \tau _0^2 + O ( \tau _0^3), \qquad \chi = \sigma _{; \alpha \beta } \dot{z}^{\alpha } \dot{z}^{\beta } =-1 + O (\tau _0^3). \end{aligned}$$

To calculate the second term in (37), let us consider the expansion of the quantity

49 $$\begin{aligned} - \kappa ^{-2} \sigma _{; \mu \alpha } u_{\nu \beta } \dot{z}^{\alpha } \dot{z}^{\beta }. \end{aligned}$$

Based on the method outlined at the beginning, the results below are obtained in agreement with^4,5^

50 $$\begin{aligned} \sigma _{; \mu \alpha } = - {\bar{g}}_{\mu \alpha } + \frac{1}{6} {\bar{g}}_{\mu }^{\delta } R^{\delta }_{\beta \alpha \gamma } \dot{\sigma }^{\beta } \sigma ^{\gamma } + O (s^2), \quad u_{\nu \beta } = (1 - \frac{1}{12} R^{\alpha \gamma } \sigma _{; \alpha } \sigma _{; \gamma } + O (s^3)) {\bar{g}}_{\nu \beta }, \end{aligned}$$

In (50), $R_{\alpha \beta \gamma \delta }$ and $R_{\alpha \beta }$ are the Riemann and Ricci tensors, respectively. From these, it then follows that

51 $$\begin{aligned} \sigma _{; \mu \alpha } \sigma _{; \alpha \beta } \dot{z}^{\alpha } \dot{z}^{\beta }{} & {} =\left( - {\bar{g}}_{\mu \alpha } + \frac{1}{6} {\bar{g}}_{\mu }^{\delta } R^{\delta }_{\beta \alpha \nu } \sigma ^{; \gamma } \sigma ^{;\beta } + O (s^2)\right) {\bar{g}}_{\nu \beta }\left( 1 - \frac{1}{12} R^{\beta ' \gamma '} \sigma _{; \beta '} \sigma _{\gamma '} + O( s^3)\right) \dot{z}^{\alpha } \dot{z}^{\beta }\nonumber \\{} & {} = \left( - {\bar{g}}_{\mu \alpha } {\bar{g}}_{\nu \beta } + \frac{1}{6} {\bar{g}}_{\mu \delta } {\bar{g}}_{\nu \beta } R^{\delta }_{\beta \alpha \gamma } \sigma ^{; \gamma } \sigma ^{; \beta } + \frac{1}{12} {\bar{g}}_{\mu \alpha } {\bar{g}}_{\nu \beta } R^{\beta ' \gamma '} \sigma _{; \beta '} \sigma _{; \gamma '}\right) \dot{z}^{\alpha } \dot{z}^{\beta }. \end{aligned}$$

Next interchange $\mu$ and $\nu$ to obtain

52 $$\begin{aligned} \sigma _{; \gamma \alpha } \sigma _{; \mu \beta } \, \dot{z}^{\alpha } \dot{z}^{\beta } = (- {\bar{g}}_{\nu \alpha } {\bar{g}}_{\mu \beta } + \frac{1}{6} {\bar{g}}_{\nu \delta } {\bar{g}}_{\mu \beta } R^{\delta }_{\beta ' \alpha \gamma } \sigma ^{; \gamma } \sigma ^{; \beta '} + \frac{1}{12} {\bar{g}}_{\nu \alpha } {\bar{g}}_{\mu \beta } R^{\beta ' \gamma '} \sigma _{; \beta '} \sigma _{; \gamma '} ) \, \dot{z}^{\alpha } \dot{z}^{\beta }. \end{aligned}$$

The difference between (51) and (52) can be computed, and it is given by

53 $$\begin{aligned} - ( \sigma _{; \mu \alpha } u_{; \nu \beta } \dot{z}^{\alpha } \dot{z}^{\beta } - \sigma _{; \nu \alpha } \sigma _{; \mu \beta } \dot{z}^{\alpha } \dot{z}^{\beta } ) = - \frac{1}{6} ( 
{\bar{g}}_{\mu \delta } {\bar{g}}_{\gamma \beta } - {\bar{g}}_{\nu \delta } {\bar{g}}_{\mu \beta }) \, R^{\delta }_{\beta \alpha \gamma } \sigma ^{; \gamma } \sigma ^{; \beta } \dot{z}^{\alpha } \dot{z}^{\beta }. \end{aligned}$$

From (46) and (47), it follows that

54 $$\begin{aligned} - \kappa ^2 ( \sigma _{; \mu \alpha } u_{\nu \beta } - \sigma _{; \nu \alpha } u_{\mu \beta } ) \dot{z}^{\alpha } \dot{z}^{\beta } = O (\tau _0). \end{aligned}$$

This shows that $- \kappa ^{-2} \sigma _{; \mu \alpha } \sigma _{\nu \beta } \dot{z}^{\alpha } \dot{z}^{\beta }$ does not contribute to (36) in the limit $\tau _0 \rightarrow 0$. Expanding the other terms, we have

55 $$\begin{aligned} \chi \kappa ^{-3} \sigma _{; \mu } u_{\nu \alpha } \dot{z}^{\alpha }{} & {} = ( -1 + O ( \tau _0^3)) \, \tau _0^{-3} \left( 1 + \frac{1}{6} \ddot{z} \tau _0^2 + \cdots \right) ^{-3} \left( - \frac{1}{2} \ddot{z}_{\mu } \tau _0^2 - \frac{1}{6} \dddot{z}_{\mu } \tau _0^3 + \cdots \right) \cdot \left( 1 - \frac{1}{12} R^{\beta \gamma } \sigma _{; \beta } \sigma _{; \gamma } \right) {\bar{g}}_{\nu \alpha } \dot{z}^{\alpha } \end{aligned}$$

56 $$\begin{aligned}{} & {} = - \tau _0^{-3} \left( - \frac{1}{2} \dddot{z}_{\mu } \tau _0^2 - \frac{1}{6} \dddot{z}_{\mu } \tau _0^3\right) {\bar{g}}_{\nu \alpha } \dot{z}^{\alpha } = \frac{1}{2} {\bar{g}}_{\mu \alpha } {\bar{g}}_{\nu \beta }\left( \ddot{z}^{\alpha } \dot{z}^{\beta } \tau _0^{-1} + \frac{1}{3} \dddot{z}^{\alpha } \dot{z}^{\beta } \right) + O (\tau _0). \end{aligned}$$

To complete the expansions, the following results are needed

57 $$\begin{aligned}{} & {} \chi \kappa ^{-3} (\sigma _{; \mu } u_{\nu \alpha } - \sigma _{; \nu } u_{\mu \alpha } ) \dot{z}^{\alpha } = \frac{1}{2} ( {\bar{g}}_{\mu \alpha } {\bar{g}}_{\nu \beta } - {\bar{g}}_{\nu \alpha } {\bar{g}}_{\mu \beta } ) ( \dot{z}^{\alpha } \ddot{z}^{\beta } \tau _0^{-1} + \frac{1}{3} z^{\alpha } \dddot{z}^{\beta }) + O ( \tau _0), \nonumber \\{} & {} \quad - \kappa ^2 ( \sigma _{; \mu } u_{\nu \alpha } - \sigma _{; \nu } u_{\mu \alpha } ) ( \ddot{z}^{\alpha } - \tilde{\kappa } \kappa ^{-1} \dot{z}^{\alpha } ) = - {\bar{g}}_{\mu \alpha } {\bar{g}}_{\nu \beta } ( \dot{z}^{\alpha } \dot{z}^{\beta } \tau _0^{-1} + O (\tau _0)), \nonumber \\{} & {} \quad - \kappa ^{-1} ( \sigma _{; \mu } u_{\nu \alpha ;\beta } -\sigma _{; \nu } u_{\mu \alpha ; \beta } ) \dot{z}^{\alpha } = - \frac{1}{2} ( {\bar{g}}_{\mu \alpha } {\bar{g}}_{\nu \beta } - {\bar{g}}_{\nu \alpha } {\bar{g}}_{\mu \beta } ) \left( \frac{1}{6} R^{\beta }_{\gamma } \dot{z}^{\alpha } \dot{z}^{\gamma } + \frac{1}{2} R_{\gamma \, \delta }^{\alpha \beta } \dot{z}^{\gamma } \dot{z}^{\delta } \right) + O ( \tau _0^{-1}),\nonumber \\{} & {} \quad - \kappa ^2 ( \sigma _{; \mu } \sigma _{\nu \alpha ;\beta } - \sigma _{; \nu } v_{\mu \alpha ; \beta } ) \dot{z}^{\alpha } \dot{z}^{\beta } = O (\tau _0), \nonumber \\{} & {} \kappa ^{-1} ( \sigma _{; \mu } v_{\nu \alpha } - \sigma _{; \nu } v_{\mu \alpha } ) \dot{z}^{\alpha } =- \frac{1}{2} {\bar{g}}_{\mu \alpha } {\bar{g}}_{\nu \beta }\left( R_{\gamma }^{\beta } - \frac{1}{6} \delta _{\gamma }^{\beta } R\right) \dot{z}^{\alpha } \dot{z}^{\gamma } + O ( \tau _0). \end{aligned}$$

Consequently, the following expansion holds to order $O (\tau _0)$

58 $$\begin{aligned} \int _{-\infty }^{\tau } \, ( v_{\mu \alpha ; \nu } - v_{\nu \alpha ; \mu } ) \dot{z}^{\alpha } \, d \tau = ( {\bar{g}}_{\mu \alpha } {\bar{g}}_{\nu \beta } - {\bar{g}}_{\nu \alpha } {\bar{g}}_{\mu \beta } ) \int _{-\infty }^{\tau } \, v^{\alpha \beta }_{\gamma } ( z ( \tau ), z (\tau ')) \dot{z}^{\gamma } (\tau ') \, d \tau ' + O (\tau _0). \end{aligned}$$

Combining these equations with (57) and (58) and substituting into (36),

59 $$\begin{aligned}{} & {} m_0 \ddot{z}_{\mu } ( \tau + \tau _0) = e {\bar{g}}^{\mu \alpha } F^{in}_{\mu \nu } ( z ( \tau + \tau _0)) z^{\nu } ( \tau + \tau _0) + e^2 ( {\bar{g}}_{\mu \beta } {\bar{g}}_{\nu \gamma } - {\bar{g}}_{\nu \beta } {\bar{g}}_{\mu \gamma } ) \dot{z} ( \tau + \tau _0) \nonumber \\{} & {} \quad \left( - \frac{1}{2} \dot{z}^{\beta } \ddot{z}^{\gamma } \tau _0^{-1} + \frac{1}{6} \dot{z}^{\beta } \dddot{z}^{\gamma } - \frac{1}{3} R^{\gamma }_{\delta } \dot{z}^{\beta } \dot{z}^{\delta } +\frac{1}{2} R_{\delta \, \eta }^{\beta \, \gamma } \dot{z}^{\delta } \dot{z}^{\eta } + \frac{1}{2} \int _{-\infty }^{\tau } \, \zeta ^{\beta \gamma }_{\delta } ( z (\tau ), z ( \tau ')) \dot{z}^{\delta } ( \tau ') \, d \tau ' +O (\tau _0) \right) . \end{aligned}$$

The Riemann tensor has disappeared because $R_{\delta \alpha \beta \eta } - R_{\delta \beta \alpha \eta }$ is skew-symmetric in $\delta , \eta$. The quantity $\zeta _{\mu \nu \alpha }$ has been introduced and is defined to be

60 $$\begin{aligned} \zeta _{\mu \nu \alpha } = v_{\mu \alpha ; \nu } - v_{\nu \alpha ; \mu }. \end{aligned}$$

The definition of parallel displacement implies that

61 $$\begin{aligned} {\bar{g}}_{\nu \beta } \, \dot{z}^{\nu } ( \tau + \tau _0) = \dot{z}_{\beta } + \ddot{z}_{\beta } \tau _0 + O ( \tau _0^2). \end{aligned}$$

The coefficients on the right hand side of this are evaluated at the proper time $\tau$. In light of (61) and the fact that ${\bar{g}}^{\mu \alpha } {\bar{g}}_{\mu \beta } = \delta _{\beta }^{\alpha }$ when (59) is multiplied by ${\bar{g}}^{\mu \alpha }$, we obtain

62 $$\begin{aligned}{} & {} m_0 {\bar{g}}^{\mu \alpha } \ddot{z}_{\mu } ( \tau + \tau _0) = e {\bar{g}}^{\mu \alpha } F^{in}_{\mu \nu } ( z ( \tau + \tau _0)) \dot{z}^{\nu } ( \tau + \tau _0) \nonumber \\{} & {} \quad + e^2\left( - \frac{1}{2} \tau _0^{-1} \dot{z}^{\alpha } - \frac{2}{3} \ddot{z}^2 \dot{z}^{\alpha } + \frac{1}{6} \dddot{z}^{\alpha } - \frac{1}{3} ( R_{\beta \gamma } \dot{z}^{\alpha } \dot{z}^{\beta } \dot{z}^{\gamma } + R^{\alpha }_{\beta } \dot{z}^{\beta } ) + \dot{z}_{\beta } \, \int _{-\infty }^{\tau } \, \zeta ^{\alpha \beta }_{\gamma } \dot{z}^{\gamma } (\tau') d \tau ' + O ( \tau _0) \right) . \end{aligned}$$

The following quantity appears in the result and needs to be simplified

63 $$\begin{aligned}{} & {} e^2 ( \dot{z}_{\gamma } + \ddot{z}_{\gamma } \tau _0+\cdots ) \left( - \frac{1}{2} \dot{z}^{\alpha } \ddot{z}^{\gamma } \tau _0^{-1} + \frac{1}{6} \dot{z}^{\alpha } \dddot{z}^{\gamma } - \frac{1}{3} R_{\delta }^{\gamma } \dot{z}^{\alpha } \dot{z}^{\delta } + \frac{1}{2} R_{\delta \, \eta }^{\alpha \, \gamma } \dot{z}^{\delta } \dot{z}^{\eta }\right. \nonumber \\{} & {} \qquad + \left. \frac{1}{2} \int _{-\infty }^{\tau } \, \zeta ^{\alpha \gamma }_{\delta } ( z (\tau ), z ( \tau ') ) \, \dot{z}^{\delta } ( \tau ') \, d \tau ' + O (\tau _0)\right) \nonumber \\{} & {} \qquad - e^2 ( \dot{z}_{\beta } + \ddot{z}_{\beta } \tau _0 + \cdots )\left( - \frac{1}{2} \dot{z}^{\beta } \ddot{z}^{\alpha } \tau _0^{-1} + \frac{1}{6} \dot{z}^{\beta } \dddot{z}^{\alpha } - \frac{1}{3} R_{\delta }^{\alpha } \, \dot{z}^{\beta } \dot{z}^{\delta } + \frac{1}{2} R_{\delta \, \eta }^{\beta \alpha } \dot{z}^{\delta } \dot{z}^{\eta }\right. \nonumber \\{} & {} \qquad +\left. \frac{1}{2} \int _{-\infty }^{\tau } \, \zeta _{\delta }^{\beta \alpha } ( z ( \tau ), z (\tau ')) \dot{z}^{\delta } ( \tau ') \, d \tau + O (\tau _0) \right) \end{aligned}$$

64 $$\begin{aligned}{} & {} \quad = e^2 \left( - \frac{1}{2} \dot{z}^{\alpha } \ddot{z}^2 - \frac{1}{2} \dot{z}^{\alpha } \ddot{z}^2 - \frac{1}{3} \dot{z}^{\gamma } R_{\delta }^{\gamma } \dot{z}^{\alpha } \dot{z}^{\beta } + 
\frac{1}{2} R_{\delta \, \eta }^{\alpha \, \gamma } \dot{z}^{\gamma } \dot{z}^{\delta } \dot{z}^{\eta } + \frac{1}{2} \dot{z}_{\gamma } \int _{-\infty }^{\tau } \, \zeta ^{\alpha \gamma }_{\delta } ( z (\tau ), z ( \tau ')) \, \dot{z}^{\delta } ( \tau ') \, d \tau '\right. \nonumber \\{} & {} \qquad - \left. \frac{1}{2} \ddot{z}^{\alpha } \tau _0^{-1} + \frac{1}{6} \dddot{z}^{\alpha } - \frac{1}{3} R^{\alpha }_{\delta } \dot{z}^{\delta } - \frac{1}{2} R^{\beta \alpha }_{\delta \, \eta } \dot{z}_{\beta } \dot{z}^{\delta } \dot{z}^{\eta } - \frac{1}{2} \dot{z}_{\beta } \int _{- \infty }^{\tau } \, \zeta ^{\beta \alpha }_{\delta } ( z ( \tau ), z (\tau ')) \dot{z}^{\delta } (\tau ') \, d \tau ' ) \right) \nonumber \\{} & {} \quad = e^2 \left( - \frac{1}{2 \tau _0} \ddot{z}^{\alpha } - \frac{2}{3} \dot{z}^{\alpha } \ddot{z}^2 + \frac{1}{6} \dddot{z}^{\alpha } - \frac{1}{3} R^{\gamma }_{\delta } \dot{z}_{\gamma } \dot{z}^{\alpha } \dot{z}^{\delta } - \frac{1}{3} R_{\delta }^{\alpha } \dot{z}^{\delta } + \dot{z}_{\beta } \int _{-\infty }^{\tau } \, \zeta ^{\alpha \beta }_{\gamma } \, (z (\tau ), z( \tau ')) \dot{z}^{\gamma }( \tau') \, d \tau ' + O ( \tau _0)\right. . \end{aligned}$$

Hence upon using (62), we finally arrive at the result

65 $$\begin{aligned}{} & {} m_0 {\bar{g}}^{\mu \alpha } \ddot{z}_{\mu } (\tau + \tau _0) = e {\bar{g}}^{\mu \alpha } F^{in}_{\mu \nu } ( z (\tau + \tau _0)) \dot{z}^{\nu } ( \tau + \tau _0) \nonumber \\{} & {} \quad + e^2 \big ( - \frac{1}{2 \tau _0} \ddot{z}^{\alpha } + \frac{2}{3} ( \ddot{z}^{\alpha } - \ddot{z}^2 \dot{z}^{\alpha }) + \frac{1}{6} \dddot{z}^{\alpha } - \frac{1}{3} ( R_{\beta \gamma } \dot{z}^{\alpha } \dot{z}^{\beta } \dot{z}^{\gamma } + R_{\beta }^{\alpha } \, \dot{z}^{\beta } ) + \dot{z}_{\beta } \int _{-\infty }^{\tau } \, \zeta ^{\alpha \beta }_{\gamma } \, \dot{z}^{\gamma } (\tau') \, d \tau ' +O ( \tau _0). \end{aligned}$$

Since the Riemann tensor is skew symmetric in the first and last indices $\delta , \eta$, the tensor $1/2 ( R_{\delta \, \gamma \eta }^{\alpha } - R_{\delta \gamma \, \eta }^{\alpha }) \dot{z}^{\delta } \dot{z}^{\eta } \dot{z}^{\gamma }$ must vanish. The term $\tau _0^{-1}$ is singular in the limit as $\tau _0$ approaches zero, but it can be absorbed in the mass by carrying out renormalization. To do so, the following procedure is adopted

$$\begin{aligned} - \frac{1}{2} \frac{e^2}{\tau _0} {\bar{g}}_{\alpha }^{\mu } \ddot{z}_{\mu } ( \tau + \tau _0) = - \frac{e^2}{2 \tau _0} \ddot{z}_{\mu } - \frac{e^2}{2} \dddot{z}_{\mu } + O (\tau _0). \end{aligned}$$

Now (65) is written out in the form

66 $$\begin{aligned}{} & {} m_0 {\bar{g}}^{\mu \alpha } \ddot{z}_{\mu } (\tau + \tau _0) = e {\bar{g}}^{\mu \alpha } F^{in}_{\mu \nu } ( z (\tau + \tau _0 )) \dot{z} ( \tau + \tau _0) - \frac{e}{2 \tau _0} {\bar{g}}_{\alpha }^{\mu } {\bar{z}} ( \tau + \tau _0) + \frac{2}{3} e^2 ( \dddot{z}^{\alpha } - \ddot{z}^2 \dot{z}^{\alpha } ) \nonumber \\{} & {} \quad - \frac{1}{2} e^2 ( R_{\beta }^{\alpha } \dot{z}^{\beta } + \dot{z}^{\alpha } R_{\beta \gamma } \dot{z}^{\beta } \dot{z}^{\gamma } ) + e^2 \dot{z}_{\beta } \int _{-\infty }^{\tau } \, \zeta ^{\alpha }_{\beta \gamma } \, \dot{z}^{\gamma } (\tau') \, d \tau ' + O ( \tau _0). \end{aligned}$$

To carry out this renormalization, it is necessary to move the singular term which contains $\tau _0^{-1}$ to the left hand side of (66), and combine it with the mass term $m_0$. To this end define the renormalized mass to be given as $m = ( m_0 + \frac{e^2}{2 \tau _0} )$. In the limit, let $\tau _0 \rightarrow 0$ such that $e^2 / \tau _0$ remains finite. Doing so, the final form for the equation of motion is obtained

67 $$\begin{aligned} m \ddot{z}^{\alpha }{} & {} = e F^{in \, \alpha }_{\beta } \, \dot{z}^{\beta } + \frac{2}{3} e^2 ( \dddot{z}^{\alpha } - \ddot{z}^2 \dot{z}^{\alpha }) - \frac{1}{3} e^2 ( R_{\beta }^{\alpha } \dot{z}^{\beta } + \dot{z}^{\alpha } R_{\beta \gamma } \dot{z}^{\beta } \dot{z}^{\gamma } ) \nonumber \\{} & {} \quad + e^2 \dot{z}^{\beta } \int _{-\infty }^{\tau } \, \zeta _{\beta \gamma }^{\alpha } \, \ddot{z}^{\gamma} (\tau') \, d \tau'. \end{aligned}$$

## Consideration of the quantum vacuum

For a charge moving inertially at nonrelativistic velocity through a static gravitational field, it has been seen that the field in a neighborhood of the charge tends to fall freely with the particle. However, a local tidal distortion due to the presence of the Riemann tensor, the net retarding force caused by this distortion is zero integrated over solid angle. Deviation of the particle’s motion when $F^{in}_{\mu \nu } =0$ is caused by a field which originates outside the classical radius. A nonlocal term comes about from a back scatter process where the Coulomb field of the particle, as it encounters bumbs in space-time, experiences jolts or hits which propagate back to the particle.

The classical analysis makes no reference to the state of the electrodynamic vacuum. There is the natural vacuum state for an extended massive body such as a neutron star. This may be thought of as a vacuum state which has come to equilibrium impressed by the action of the gravitational field. There is the Hartle-Hawking vacuum which corresponds to the natural vacuum of a black hole in a tiny neighborhood of it and corresponds to a black hole in equilibrium with a bath of blackbody radiation. Finally, there is the Unruh vacuum state, which would be related to a black hole produced by collapse of a star.

It may be thought that in the Hawking–Hartle vacuum, a charge fixed in the gravitational field of a black hole hence accelarated should not be able to extract energy from the freely falling vacuum fluctuations and radiate contrary to the classical result. This does not occur due to the fact that in the Hawking–Hartle vacuum, the fluctuations are distributed with a thermal spectrum so that there can be no systematic exchange of energy between charge and field. It seems in all these cases, the classical result regarding radiation emitted and radiative-reaction force on an electron in the various states of motion can be understood in terms of the spectrum of the field fluctuations apparent to the charge. The classical results could have been incorrect as the quantum equations differ from the classical by terms going like Planck’s constant or classical results for a type of motion only apply to just one of the three vacuum states but not to all. If there is duality between the radiation-reaction and vacuum-fluctuation picture that the spectrum of the field fluctuations for a given motion and vacuum must accord with classical results. Thinking in terms of the Heisenberg equations make no reference to the state of the field, classical results have to apply with equal validity each of these distict vacuua, and this provides a connection to the equivalence principle. Also the decay of an atom can be viewed in terms of the electromagnetic field or as the consequence of the radiative self force of the electron, a link with the equivalence principle could be asserted.

## Summary

An accelerated charged particle should emit radiation and hence suffer a reactive damping force, no matter what the nature of the acceleration. The equivalence principle forces an element of vagueness and uncertainty into the problem. Even though this work does not explicitly bring in quantum mechanics, it suggets that the observation of such radiation could imply new physics might be deduced with regard to spacetime. It would be of interest to see the results of experiments done according to the setup outlined here. The integral term appearing in (67) might reveal further information as to the physical nature of spacetime.

## Data Availability

All data generated or analysed during this study are included in this published article.
